# Micro-CT evaluation of internal adaptation in composite restorations using incremental and sonic-activated techniques

**DOI:** 10.6026/973206300222539

**Published:** 2026-04-30

**Authors:** Parthivi Singh, Vartul Dwivedi, Siddhi Yadav, Keerti Mishra, Manika Gautam, Tanushri Pandey

**Affiliations:** 1 Department of Conservative Dentistry and Endodontics, Peoples Pental Academy, Peoples University, Bhopal, Madhya Pradesh, India; 2 Department of Conservative Dentistry and Endodontics, People's College of Dental sciences and Research Centre, Bhopal, Madhya Pradesh, India; 3 Department of Conservative Dentistry and Endodontics, District Hospital, Indore, Madhya Pradesh, India; 4 Department of Conservative dentistry and Endodontics, District Hospital, Sehore, Bhopal, Madhya Pradesh, India

**Keywords:** Micro-CT, internal adaptation, composite restoration, sonic-activation, incremental technique, internal voids

## Abstract

Internal adaptation of composite restorations remains a challenge, particularly in posterior cavities, as voids and gaps can compromise clinical longevity. Therefore, it is of interest to compare the internal adaptation of composite restorations placed using conventional incremental, oblique incremental and sonic-activated bulk-fill techniques using high-resolution micro-computed tomography. Sixty standardized Class II cavities were restored (n=20 per group) and evaluated for internal gap volume, void volume and total defect percentage. The sonic- activated bulk-fill technique demonstrated significantly lower internal gap and void volumes compared to both incremental techniques (p<0.001). Sonic-activation provides superior internal adaptation, suggesting improved clinical performance over conventional layering methods.

## Background:

The success of direct composite restorations in the posterior teeth hinges on the complex nature of interaction between material properties, quality of adhesive interface and the methodology in use during their placement. The internal adaptation, the proximity of the restorative material with the prepared cavity walls became one of these parameters, which has proven to be a critical parameter that directly determines the mechanical performance, marginal integrity and biological compatibility of the restoration [1]. It is poor internal adaptation that appears as interfacial gaps and intra body voids that undermine stress distribution, where cracks can form and grow and potentially predispose the restoration to premature failure [2]. The reason is that composite resin is the most popular restorative material that is used as a direct restoration on the posterior tooth as this type of restorative material has good esthetic qualities, low carving design needs and its mechanical properties are continually being enhanced. Nonetheless, the natural polymerization contraction of resin-based composite, which is normally between 1.5 to 5 percent by volume forms contraction stresses at bonded interface during curing that may sever the adhesive seal and form internal gaps [3]. These stresses that arise because of polymerization depend on the cavity geometry, the configuration factor (C- factor), material formulation and the placement method [4]. Incremental layering technique is the gold standard method of placing restorations of the posterior composite restorations over a number of decades. This method attempts to minimize the amount of material undergoing polymerization at a particular time, in order to decrease the overall compressive stress of net polymerization stress at each bonded increment, by stacking composite in successive layers of 2 mm or less [5]. A number of layering strategies have been promoted such as horizontal, oblique and centripetal incremental strategies, each alleging to possess benefits in terms of stress management and the quality of adaptation [6]. An oblique layering technique which is specifically proposed has been broadly advocated owing to the fact that it diminishes the C-factor by ensuring every increment is bonded to fewer cavity walls, theoretically, reducing interfacial stress and enhancing adaptation [7]. Even through the theoretical benefits of incremental layering are no longer doubted, the methodology does not lack limitations. Both layers present the risk of entrapment of voids between increments, contamination of the interfaces between layers and the incomplete accommodation of the cavity walls, especially in locations with difficult access such as the gingival floor of proximal boxes [8]. The incremental technique is operator-dependent, which implies that clinical outcomes are very diverse and largely dependent on skill and carefulness of the practitioner [9]. Moreover, time-consuming character of the process of inserting several increments raises the risk of contamination of moisture in posterior cavities in which isolation can be undermined. A series of shortcomings were overcome by the introduction of sonic-activated composite placement systems. The sonicFill system was initially developed by Kerr Corporation and it involves the use of a specially formulated composite resin that experiences a radical drop in viscosity when exposed to sonic energy when dispensing. This rheological adjustment enables the material to flow easily and conform easily to the walls of the cavities and internal angles during installation. When sonic energy is stopped, the material is reverted to a non-slumping, sculptable material suitable in making anatomical contouring [10]. The capability to insert this material at the same rate of up to 5 mm deep without loss of depth of cure or quality of adaptation is a big break in the traditional placement philosophy [11]. Historically, the two-dimensional techniques including cross-sectional microscopy, dye penetration evaluation and interfacial gap measurement at a few points have been used to evaluate internal adaptation. Although these techniques have gathered useful data, the techniques can only give information at selected sectioning planes and they do not represent the three-dimensional nature of the tooth-restoration interface [12]. Micro-computed tomography has become the new standard in the evaluation of internal adaptation, where it is possible to visualize and quantify interfacial gaps and internal voids in the entire volume of the restoration in a nondestructive and three-dimensional manner [13]. This technology offers isotropic spatial resolution on the micrometer scale, which is capable of measuring volumetric defects in the adaptation process accurately that would not have been possible to measure or even identify by any other means [14]. A number of studies have been carried out to assess different factors of composite restorations using micro-CT, such as marginal adaptation, polymerization shrinkage and analysis of voids. Nevertheless, three-dimensional comparisons explicitly focused on the internal adaptation of restorations, which are placed using sonic-activated techniques and conventional and oblique incremental techniques using the current- generation materials, are scarce in the literature [15]. Moreover, independent quantitative volumetric study of interfacial gaps and intrabody in separate entities has not been studied in depth, even though the clinical implication of each form of defect is different [16]. Therefore, it is of interest to assess and compare internal adaptation of Class II composite restorations installed by three methods conventional horizontal incremental, oblique incremental and sonic-activated bulk fill by quantitative 3D micro-CT analysis.

## Materials and Methods:

This comparative study is an *in vitro* study performed at the advanced imaging and biomaterials research laboratory with respect to the use of extracted human teeth with the use of the institutional ethical approval. Sixty full-size intact human first and second molars were collected and put in 0.1% thymol solution maintained at 4°C and at most 3 months. The patients aged between 20 and 50 years had their first and second molars extracted due to periodontal or surgical reasons.

## Inclusion criteria:

Owing to sound mandibular molars with well-developed roots, free of caries, cracks, restorations, or developmental anomalies and not exceeding normal anatomical range in terms of the width of the cusp mesiodistal (10.0-12.0 mm) and buccolingual (9.5-11.5 mm) width.

## Exclusion criteria:

Teeth that showed visible fracture lines, carious lesion, existing restorations, hypomineralization defects, root resorption, or attrition above the enamel. All teeth were examined under a stereomicroscope using the 10x magnification and pre-scanned using micro-CT to rule out any internal faults in the specimen. Cavity Preparation Each tooth was cemented in a special acrylic resin block leaving the coronal part open. Unilateral preparation of standardized Class II mesio-occlusal (MO) cavity preparations was done by one skilled operator using a high-speed handpiece (W & H Alegra, W & H Dentalwerk, Bueermoos, Austria) with abundant water irrigation. Initial preparation was done with cylindrical diamond burs (ISO #835-012, medium grit, Komet, Lemgo, Germany) and final finishing was done on fine-grit burs.

## Preparatory phase:

Occlusal isthmus width- 3.5 mm (about one-third of the intercuspal distance), pulpal floor depth- 3.5 mm at the cusp tip, proximal box- 1 mm above the cementoenamel junction at axiogingival line angle and gingival seat- 1.5 mm. There was a 6-degree occlusal divergence in cavity walls. After each five preparations, the bur was changed. The dimensions of all dimensions were checked using a UNC-15 periodontal probe and digital calipers (Mitutoyo, Kawasaki, Japan). Specimens were positioned with a sectional matrix system (Palodent V3, Dentsply Sirona, Charlotte, NC, USA) with a corresponding wedge and separation ring to hold the proximal box together during restoration. The 60 ready teeth were randomly assigned 3 sets of 20 specimens each.

## Computer generated random array:

Group 1 (Conventional Incremental, n =20): Filtek Z350 XT (3M, St. Paul, MN, USA), horizontal incremental method. As shown below, this was the condition in Group 2 (Oblique Incremental, n=20): Filtek Z350 XT (3M), oblique incremental technique. Group 3 (Sonic- Activated Bulk Fill, n= 20): SonicFill 3 (Kerr Corporation, Orange, CA, USA), sonic-activated method.

## Adhesive protocol:

Unified adhesive protocol was implemented in all the groups. The surface of enamels and dentin were etched using 37 percent phosphoric acid gel (Scotchbond Universal Etchant, 3M) of 15 seconds on dentin and 30 seconds on enamel, rinsed with plenty of water of 15 seconds and blot-dried with cotton pellets to keep dentin moist. Two coats of a universal adhesive (Scotchbond Universal Plus, 3M) were applied in 20-second bursts with active agitation and gently air-thinned in 5-second bursts and light-cured in 20-second bursts with an LED curing unit (Elipar DeepCure-S, 3M) confirmed to have an irradiance output of 1,470 mW/cm^2^.

## Restorative procedures:

Group 1 (Conventional Incremental): Filtek Z350 XT composite (shade A2 Body) was laid in horizontal layers of about 2 mm of thickness, each. The initial increment was done at the gingival floor of the proximal box and fitted using a composite placement instrument (DERA, Zeffiro, Italy). The layers were light-cured in sets of 20 seconds and another layer was placed. Each restoration took around four or five increments to complete. Group 2 (Oblique Incremental): Oblique layering strategy was used to place the same composite material (Filtek Z350 XT shade A2 Body). The initial increment was laid as a wedge-shaped layer against the gingival floor and one wall of the proximal box and oblique increments that followed each other in turn towards buccal and lingual walls respectively. Every increment was left to dry and was light-cured in 20 seconds. Each restoration needed about four to be done. Group 3 (Sonic-Activated Bulk Fill): SonicFill 3 composite (shade A2) was placed directly into the cavity preparation by use of SonicFill handpiece (KaVo Kerr) coupled and attached to a compatible dental handpiece, operating at sonic frequency intensity level (3 of 5). It was dispensed to start at the gingival floor of the proximal box and the tip kept inside the dispensed material in order to reduce entrapment of air. One increment of bulk that was up to 5 mm was put in place and fitted to the cavity walls in the active sonic dispensing stage. Once the sonic energy was stopped, the material was shaping slightly with a composite instrument to an anatomical form. The light curing was done 40 seconds of the occlusal surface and 20 seconds of the buccal surface. All the restorations were completed with fine and superfine aluminum oxide discs (Sof-Lex, 3M) and diamond finishing burs (Komet). The final increment was cured and then the sectional matrix and ring were removed. Before scanning, samples were placed in distilled water at 37°C and allowed to stay in this solution during 48 hours. 3.6 Micro-CT Scanning Protocol. The high-resolution micro-CT scanner (SkyScan 1275, Bruker, Kontich, Belgium) was used to scan all specimens with the following scan parameters: X-ray source voltage of 100 kV, beam current of 100 µA, filtration of aluminum and copper (Al 1.0 mm + Cu 0.05 mm), 360 degrees rotation step of 0.3 degrees, frame averaging of 4 and isotropic voxel size of the 12 µm. The average scan time of each specimen was 45 minutes. Before each scanning the correction of flat-field was done. Image reconstruction and analysis are essential parts of the research, which will be discussed in this section. NRecon software (version 1.7.4, Bruker) was used to reconstruct raw projection images into cross-sectional slices with standard reconstruction parameters, such as ring artifact correction (level 8), beam hardening correction (40%) and uniform density thresholding. CTAn software was used, which is a 3D analysis (version 1.20, Bruker) and volumetric rendering with CTVox software (Bruker).

## Segmentation-based analysis was used to measure the following parameters:

[1] Internal gap volume (mm3): This is the gap space that exists between the composite restoration and cavity wall (tooth-composite interface), which indicates defects in adaptation.

[2] Internal void volume (mm3): This is defined as enclosed porosities within the body of the composite restoration that are not related to the external surface or to the tooth-composite interface and represents air inclusions or interlayer voids.

[3] Total restoration volume (mm3): This is the total amount of volume of the composite restoration.

[4] Total defect percentage ( ) percent: = [(internal gap volume + internal void volume)/total restoration volume) x 100].

[5] Segmentation was carried out through the global thresholding depending on the unique attenuation coefficients of enamel, dentin, composite resin and air/ void spaces. The thresholding values were adjusted with reference scans of known materials and checked on a subset of the specimens by two independent observers. An area of interest that included the whole restoration and a 50-µm perimeter around the tooth-composite interface was selected in each specimen.

## Reliability assessment:

Intra-examiner reliability was also evaluated by repeating the examination on 10 randomly chosen specimen after two weeks. Inter- examiner reliability was also tested by making a second trained observer analyze the same 10 specimens independently. Intraclass correlation coefficients (ICC) were obtained.

## Statistical analysis:

Data were analyzed using SPSS version 28.0 (IBM Corp., Armonk, NY, USA). Normality was assessed using the Shapiro-Wilk test and homogeneity of variances was verified with Levene's test. One-way analysis of variance (ANOVA) was used to compare continuous outcome variables among the three groups, followed by Tukey's honestly significant difference (HSD) post-hoc test for pairwise comparisons. The significance level was set at α = 0.05.

## Results:

Intra-examiner and inter-examiner reliability were excellent for all measured parameters. The ICC values for internal gap volume were 0.962 and 0.941, respectively, while those for internal void volume were 0.955 and 0.933, confirming high reproducibility and consistency of the micro-CT assessment protocol. One-way ANOVA showed statistically significant differences among the three restorative techniques for internal gap volume, internal void volume and total defect percentage (p<0.001). The sonic-activated bulk-fill group showed the most favorable internal adaptation, with the lowest internal gap volume, internal void volume and total defect percentage. The conventional incremental group showed the highest defect values, while the oblique incremental group showed intermediate results. However, total restoration volume did not differ significantly among the groups (p=0.667), as shown in Table 1 (see PDF). Regional evaluation of internal gap volume demonstrated significant differences at all cavity regions. The gingival floor showed the highest gap volume in all groups, indicating it was the most critical region for adaptation. The sonic-activated group showed the greatest reduction in gap volume, particularly at the gingival floor, followed by the axial wall, pulpal floor and buccal/lingual walls. These findings are presented in Table 2 (see PDF). Internal void analysis revealed that the conventional incremental group had the highest number of voids, including a greater proportion of medium and large voids. The sonic-activated group had the lowest total number of voids, with most defects limited to the small void category. The oblique incremental group showed intermediate values. The distribution of void sizes is shown in Table 3 (see PDF). Three- dimensional micro-CT renderings supported the quantitative findings. The conventional incremental group showed multiple scattered voids, especially at interlayer interfaces. The oblique incremental group showed fewer voids, mainly along the gingival floor and oblique increment margins. In contrast, the sonic-activated group demonstrated a more homogeneous internal structure with minimal void formation and improved wall adaptation.

## Discussion:

The findings of the present research established that the sonic-activated bulk fill method yielded considerably high quality of internal adaptation as compared to the traditional and incremental techniques of composite restorations placement, when using Class II composite restorations. The null hypothesis was then rejected in all the comparisons which were done between the sonic-activated group and the majority of the comparisons between the two incremental approaches. The high level of internal adaptation that is possible with SonicFill 3 system that operates sonically can be ascribed to the rheological peculiarities that this material demonstrates during its placement. Based on the dispensing processes, the modifiers that are incorporated in the composite matrix react by decreasing the viscosity of the material, which was roughly 250 Pa/s, to less than 3 Pa/s when sonic energy is applied, allowing the flowing and capability of the composite to conform to the walls of cavity structures [17]. This is because dramatic viscosity leads to easier penetration into the undercuts, internal angles and the difficult gingival floor area of proximal boxes where the conventional composites are usually unable to adapt all the way [18]. When sonic energy is stopped, the rapid viscosity recovery is used so that the material does not slump in its position of placement, which is essential in the posterior cavity designs [19]. Clinically, the much smaller volumes of internal gap that were seen at the gingival floor in the sonic-activation group are also very interesting. The gingival floor of Class II restorations is globally known to be the most susceptible area to deficits in adaptation because of its geometrical complexity, poor accessibility of instruments manipulation and difficulty of determining full material adaptation in clinical conditions [20]. The capacity of the sonic-activated material to flow directly into this region during sonic vibration displacing air and making sure that the walls are in intimate contact with each other is a significant clinical benefit over the manual methods of packing [21]. Among all groups, the highest values of internal gap volumes and void counts were determined by the conventional horizontal incremental technique. The observation is consistent with other studies carried out in the past, which have brought about the interlayer interface as a major source of porosity in incrementally placed composite restorations [22]. Any interface between successive increments is a potential location of air entrapment and the condensing and adapting of each increment to the underlying cured surface inevitably introduces microporosities which grow with the number of increments [23]. The greater number of increments needed in this technique (four to five layers) multiplicatively adds to the chances of forming defects. The oblique incremental method gave average results and it was better than the traditional method but not better than a sonic-activated method. Enhanced adaptation of the oblique method in lieu of horizontal layering is in line with the known fact that the oblique increments decrease the C-factor due to bonding to fewer opposing cavity walls at once [24]. This decrease in configuration factor corresponds to reduced polymerization shrinkage stress at each increment-wall interface thus decreasing the size of contraction gaps. To add to that, the oblique orientation of every increment can provide the option of a better adaptation by enabling the composite to be packed against individual cavity walls instead of trying to be adapted to opposing walls [25]. The analysis of the void size distribution showed significant data regarding the type of defects related to each method. The standard incremental group had a larger total number of voids, as well as a much higher percentage of medium and large ones. These larger vacuoles have more critical clinical implications compared to small porosities since they can act as centres of increased stress concentration and can act as a site of fatigue crack propagation in cyclic loading of the occlusal teeth [26]. The small voids prevailing in the sonic-activated group indicate that the small porosities that do exist must be by chance of the dispensing process instead of systematic sites of defects [27]. The micro-CT as the assessment method used in this research had a number of benefits in comparison to the traditional methods of assessment. In contrast to cross-sectional techniques, which damage the specimen and give information at the sectioned plane of the sample, micro-CT allows the whole tooth-restoration interface of the tooth to be studied in three dimensions without damaging the sample [28]. This is necessary to identify faults that can be discontinuous in the restoration and would not be encountered by random sectioning methods. The voxel resolution of 12 µm that is employed in this study is appropriate to identify clinically significant defects of adaptations but with manageable scan time and data volumes [29]. The process of clinical significance of the differences of adoption that were noticed in this study should be viewed in a larger scope of restoration longevity. The internal gaps between the tooth and the restoration allow oral fluid percolation, bacterial spread and enzymatic breakdown of the adhesive layer, leading to the secondary caries and secondary failure of the restoration [30]. In the same vein, internal porosity in the restoration body diminishes its effective cross-sectional area, fracture resistance and potentially may be clinically evident in the form of bulk fracture during loading [31]. Although the absolute volumes of defects present in each and every group were fairly low when proportional to total restoration volume (0.201% to 0.460%), the collective impact of these defects through the years of clinical service coupled with continuous hydrolytic and mechanical corrosion and erosion can have a huge effect on the survival of restoration in the long term [32]. One should take into account the fact that sonic- activated technique utilizes a specially designed compound material (SonicFill 3) as opposed to the use of a different approach to placement of a standard composite. The performance of SonicFill 3, its high filler loading (81.3 percent by weight) and sonic-responsive modifier system which is part of the material composition are all interrelated and inseparable with the placement technique [33]. Hence, the differences in internal adaptation that are observed are the joint influence of material formulation and placement technique and not technique. Subsequent research on the comparison of sonic activation of conventional and sonic-specific fill materials would aid in the limitation of the relative importance of each factor. The internal validity of this study is enhanced by the standardization of cavity preparations, adhesive protocols and also condition of the operator. Nevertheless, a number of limitations have to be admitted. The *in vitro* design fails to consider the difficulties of clinical placement, such as patient motion, restricted access, water pollution and time constraints that can enhance technique-based changes in the quality of adaptation [34]. It was determined at one instant without aging and simulation of loading and the effect of thermomechanical cycling on the adaptation differences observed is yet to be explored [35]. Also, it is possible that the results of the study cannot be directly extended to other cavity designs like Class I or massive MOD preparations since only one cavity design (Class II MO) was studied. Although the single-operator design is consistent, it can impair the external validity to clinical practice where operator variability is a major consideration. The results of the incremental technique, especially, will tend to be more varied between practitioners of varying levels of experience and, therefore, will tend to increase the benefits of the less technique-sensitive sonic-activated method on practice in clinical settings [36].

## Conclusion:

The internal adaptation of the sonic-activated bulk fill and no interfacial gaps and intra body voids are many times better in placing the Class II restorations than the conventional and oblique incremental techniques. The highest improvement was noticed in the area of critical gingival edge with the sonic-activated technique recording the closest adaptation of the wall. These results indicate that sonic- activated bulk fill systems are clinically beneficial as an alternative to incremental layering due to their ability to provide optimal internal preparation in posterior composite restorations.
